# 6-week cryo-compression therapy and opioid use after unicompartmental knee arthroplasty with comparable pain scores: a secondary analysis of a randomized controlled trial

**DOI:** 10.2340/17453674.2026.45963

**Published:** 2026-06-10

**Authors:** Hannah J BIEMANS, Astrid J DE VRIES, Reinoud W BROUWER

**Affiliations:** Orthopedic Surgery Department, Martini Hospital, Groningen, the Netherlands

## Abstract

**Background and purpose:**

Cryo-compression benefits after knee arthroplasty appear short-lived. Evidence in unicompartmental knee arthroplasty (UKA) is particularly scarce. We aimed to assess 6-week cryo-compression on pain (including oxycodone consumption), function, and quality of life (QoL) at 6 weeks following UKA compared with standard care.

**Methods:**

In this single-center, single-blind randomized controlled trial (NCT05572359), adults scheduled for UKA were randomly assigned (1:1) to the regular or cryo-compression group. The cryo-compression group was instructed to use the cold compression brace 5 times daily for 6 weeks. Primary outcome (Numeric Rating Scale [NRS] pain at rest) and secondary outcomes (NRS pain during loading, Knee Injury and Osteoarthritis Outcome Score-subscales, Oxford Knee Score, Work, Osteoarthritis and joint-Replacement Questionnaire [WORQ], physical tests, EuroQol 5-Dimensions 5-Level [EQ5D-5L]) were measured preoperatively and at 6 weeks. Oxycodone consumption was recorded daily.

**Results:**

At 6 weeks, there was no between-group difference in pain at rest (mean difference 0.1 points, CI –0.6 to 0.9). The cryo-compression group consumed 46% less oxycodone over the 6 weeks (CI 9–68), with an absolute median difference of 3.5 5-mg tablets in total. WORQ and EQ5D-5L index-scores improved more in the cryo-compression group (8.7 points, CI 0.7–16.6, and 0.10 points, CI 0.03–0.2, respectively), but neither reached the minimal clinically important difference (13 and 0.11).

**Conclusion:**

6-week cryo-compression following UKA demonstrated no differences in pain at rest or functional and QoL-related outcomes. Oxycodone consumption was lower in the cryo-compression group, although absolute differences were small.

Unicompartmental knee arthroplasty (UKA) is a well-established treatment for isolated compartment osteoarthritis, providing significant improvements in pain relief and knee function [1]. However, postoperative pain, opioid consumption, and functional limitations remain challenges during the initial weeks [2-3].

Cryotherapy (+ compression) is frequently studied as an adjunct to postoperative care because of its potential benefits for pain control, opioid consumption, and knee function [4-11]. However, these benefits seem to be limited to the first postoperative week, potentially because most studies limit the intervention period to this initial week [4-8]. Only a few studies to date have used a longer intervention period of 2 weeks, and although these randomized controlled trials (RCTs) demonstrate benefits at 2 weeks, the effects appear to diminish after this period [9-11].

Despite the growing popularity of UKA, most cryo-compression research has focused on total knee arthroplasty (TKA), leaving a gap in evidence for UKA [4-6,9-11]. Although opioid use is less common after UKA than after TKA, approximately 8% of UKA patients still use opioids 3 to 6 months postoperatively [12-13]. After 1 year, up to 23% of UKA patients report chronic pain, and 1.5% continue to use opioids because of knee pain [14]. This underscores the clinical relevance of interventions aimed at reducing opioid use in this population as well. Only 2 studies on cryo-compression are known to have included UKA patients [7-8].

Our primary aim is to evaluate the effects of 6-week cryo-compression on pain at rest following UKA compared with standard care. Secondary outcomes are pain—other than pain at rest—including opioid consumption, function, quality of life (QoL), patient satisfaction, protocol compliance, brace-related discomfort, and complications. The hypothesis is that extending cryo-compression to 6 weeks may result in reduced pain at 6 weeks following UKA.

## Methods

### Study design

This single-center, single-blind, parallel-group RCT was conducted in the Orthopedic Surgery Department at a Dutch peripheral teaching hospital. The study protocol has been published [15]. This study specifically focuses on the 6-week outcomes and oxycodone consumption during this period in UKA patients. The study adhered to the Consolidated Standards Of Reporting Trials (CONSORT) guidelines [16].

### Participants

Adults aged 18 years or older, scheduled for UKA due to end-stage osteoarthritis, were eligible for inclusion. The exclusion criteria were intraoperative conversion to TKA, rheumatoid arthritis, comorbidities potentially interfering with cryo-compression (as judged by the orthopedic surgeon), inability to read Dutch, and lack of access to a freezer. Orthopedic surgeons informed eligible participants about the study. Written informed consent was obtained from all participants prior to any study procedures.

### Randomization and blinding

Participants were randomly assigned to the control group (regular group) or the intervention group (cryo-compression group) using block randomization with a 1:1 allocation ratio. Allocation concealment was achieved using sequentially numbered, opaque, sealed envelopes. The allocation sequence was computer-generated, and allocation occurred after baseline measurements to maintain assessor blinding. Blinded assessors conducted follow-up measurements.

### Intervention

The cryo-compression group received instructions regarding the intervention using the U-Sport Ultimate Recover Knee Cold Compression Brace [17], which includes a cold pack that must be stored in a freezer between sessions. They were instructed to use the brace 5 times daily up to 20 min per session for 6 weeks, starting after hospital discharge. This totaled 42 days and 210 sessions of cryo-compression. Compression pressure could be adjusted using a hand pump, allowing for personalization of pressure.

Both groups received standardized care during hospitalization, following the hospital’s rapid recovery protocol. This included early full weightbearing mobilization, range of motion (ROM) exercises supervised by a physiotherapist, cold packs application, and 24-h postoperative compressive bandaging. Pain management followed standard protocol, which included paracetamol, non-steroidal anti-inflammatory drugs (NSAIDs) when tolerated, and oxycodone 10 mg. Participants were instructed to taper oxycodone 10 mg use as pain allowed. Additionally, escape doses of oxycodone 5 mg could be taken up to 4 times daily for breakthrough pain. Cold packs were permitted as part of standard care during hospitalization and after discharge.

After discharge, participants were instructed to complete a daily log for 6 weeks, recording the frequency of cold pack (regular group) or cold compression brace (cryo-compression group) use in days and sessions (including compression), along with oxycodone consumption (5 and 10 mg).

### Baseline characteristics

Baseline characteristics, including sex (men/women), age, body mass index, American Society of Anesthesiologists (ASA) score (I–IV), knee compartment (medial/lateral), Kellgren-Lawrence score (0–4), contralateral TKA/UKA (yes/no), analgesic schedule (normal/no NSAIDs/other), and length of hospital stay (hours), were obtained from medical charts.

### Outcome measures

#### Primary outcome

The primary outcome was perceived pain at rest at 6 weeks (adjusted for preoperative NRS pain at rest). Pain at rest was measured using the 11-point Numerical Rating Scale (NRS), where 0 indicated no pain and 10 indicated unbearable pain [18].

#### Secondary outcomes

Pain other than pain at rest was assessed using the 11-point NRS for pain during loading [18], and the Knee injury and Osteoarthritis Outcome Score (KOOS) pain and symptoms subscales preoperatively and at 6 weeks. Each KOOS subscale score ranges from 0 (worst) to 100 (best) [19]. Furthermore, oxycodone consumption (5 and 10 mg) and the number of participants using oxycodone were extracted from the daily logs (total and weekly). Participants who did not report their oxycodone consumption data were contacted by phone. At 6 weeks, an anchor question was used to assess self-reported perceived improvement in pain, using a 7-point Likert scale (very much improved to very much worsened).

Functional outcomes were assessed using the KOOS Activities of daily living (ADL) [19], the Oxford Knee Score (OKS), and the Work, Osteoarthritis and Joint-Replacement Questionnaire (WORQ) preoperatively and at 6 weeks. The OKS measures function and pain during ADL on a 12 (best) to 60 (worst) scale [20]. The WORQ uses a scale from 0 (worst) to 100 (best) to measure work-related physical difficulties [21]. Additionally, 3 physical performance tests were conducted preoperatively and at 6 weeks. ROM was determined in degrees [22]. Knee circumference was measured in centimeters (cm) at the mid-patellar level and 7 cm proximal and distal as a reliable indicator of swelling [22]. The Timed Up and Go (TUG) test assessed function by measuring the time (seconds) required to stand, walk 3 meters, return to the chair, and sit down [23]. An anchor question of daily functioning was used to assess self-reported perceived improvement at 6 weeks (7-point Likert scale).

QoL was assessed using the KOOS-QoL [19] and the EuroQol-5 Dimension-5 Levels (EQ5D-5L) preoperatively and at 6 weeks. The EQ5D-5L measures health across 5 dimensions: mobility, self-care, usual activities, pain/discomfort, and anxiety/depression [24]. It provides an index score (ranging from <0 to 1) and a Visual Analogue Scale (VAS, 0 to 100), with higher scores indicating better health. The index score was calculated using the Dutch tariff described by Versteegh et al. [25].

Patient satisfaction with the UKA and the brace (cryo-compression group) was measured at 6 weeks using an 11-point NRS, where 0 indicated no satisfaction and 10 indicated complete satisfaction. Data on complications after hospital discharge related to UKA was obtained from medical records at 6 weeks. Brace-related discomfort was recorded using a yes/no question, with further specification if discomfort was reported.

Protocol compliance in the cryo-compression group was based on participants’ daily logs and measured as the number of completed cryotherapy days, cryotherapy sessions, and compression sessions, relative to the total instructed (42, 210, and 210, respectively). Cryotherapy frequency using cold packs was measured in days and sessions for the regular group.

### Sample size

The sample size was calculated based on a 6-week pain-at-rest score of 3.2 (standard deviation [SD] 2.4) in TKA patients [5]. A minimal clinically important difference (MCID) of 1.4 was used [26]. Two-sided testing, a power of 80%, and an alpha of 0.05 led to a sample of 47 patients per group. In case of a dropout, all data gathered until that moment was used. This trial [15] was registered for both UKA and TKA patient populations combined (estimated number of patients required = 208); the smaller sample size in this study reflects the focus on the UKA population alone.

### Statistics

Descriptive statistics summarized continuous variables as means with SDs for normally distributed data, and medians with interquartile ranges (IQRs) for non-normally distributed data. Categorical variables were reported as frequencies (n).

Analyses for the primary outcome and most secondary outcomes followed the intention-to-treat principle [27], including all randomized participants regardless of adherence to the cryo-compression protocol or missing follow-up data. Missing data was assumed to be missing at random. The primary outcome, NRS pain at rest at 6 weeks, was analyzed using an analysis of covariance (ANCOVA) to assess group differences, adjusting for baseline NRS pain at rest. Estimated marginal means and between-group difference with 95% confidence intervals (CI) were presented to provide the magnitude and precision of the treatment effect. Secondary outcomes measured at 2 time points were analyzed using Generalized Estimating Equations (GEE) with an exchangeable data structure to assess the between-group difference in changes from baseline to 6 weeks. This model included group (regular group vs cryo-compression group), time (preoperative vs 6 weeks), and their interaction (time × group). The use of GEE allowed us to retain all participants with at least 1 available outcome measurement, in line with the intention-to-treat-principle. To evaluate the impact of missing data for the GEE outcomes, a sensitivity analysis was conducted using a complete-case subset. The exchangeable data structure was chosen to account for within-subject correlation between repeated measurements. Between-group differences were presented with CI. Data on anchor questions, satisfaction, and complications was analyzed using an independent t-test for normally distributed continuous data, a Mann–Whitney U-test for non-normally distributed continuous data, and a chi-square test for categorical variables.

For the secondary objectives of oxycodone consumption, cryo-compression frequencies, and protocol compliance, the intention-to-treat principle was not applied. To ensure reliable estimation, only patients with ≥ 75% completed entries (or entries retrieved by telephone) were included, representing a complete-case subset. Oxycodone consumption results were presented as medians of 5 mg and 10 mg tablets consumed with IQRs, as well as the number of participants using oxycodone per week and in total during the 6 weeks. Linear regression was used to estimate the effect of group allocation on oxycodone 5 mg and 10 mg consumption. To improve precision, analgesic schedule (normal/no NSAIDs) and sex (men/women) were included as covariates based on the literature [28-29]. For oxycodone 10 mg consumption, length of hospital stay (hours) was also included, as patients routinely received oxycodone 10 mg twice a day during hospitalization. Because oxycodone consumption data was skewed, log10 transformations were applied to meet assumptions; estimates were back-transformed to provide the percentage difference between groups with CI. Cryo-compression frequencies were analyzed using independent t-tests or Mann–Whitney U-tests, depending on data distribution.

Data was analyzed using IBM SPSS Statistics version 25 (IBM Corp, Armonk, NY, USA), with two-sided tests and a significance level of < 0.05.

### Ethics, data sharing, funding, and disclosures

The local medical ethics committee MEC-U approved this study (R22.095/NL-number NL81956.100.22). All participants provided written informed consent in accordance with the Declaration of Helsinki and Good Clinical Practice regulations. Personal data was handled in agreement with the Dutch Personal Data Protection Act (AGV). The trial was registered at ClinicalTrials.gov (NCT05572359). We aim to facilitate data-sharing in line with the Findability, Accessibility, Interoperability and Reuse (FAIR) principles, and reasonable data-sharing requests will be considered. Funding was received from the Dutch Arthroplasty Register (LROI) (grant number LROI-RG 2023-004). The LROI was not involved in the study design; the collection, management, analysis and interpretation of data; writing of the report; or the decision to submit the report. The authors declare no relevant conflicts of interest related to this study. Complete disclosure of interest forms according to ICMJE are available on the article page, doi: 10.2340/17453674.2026.45963

**Figure:**
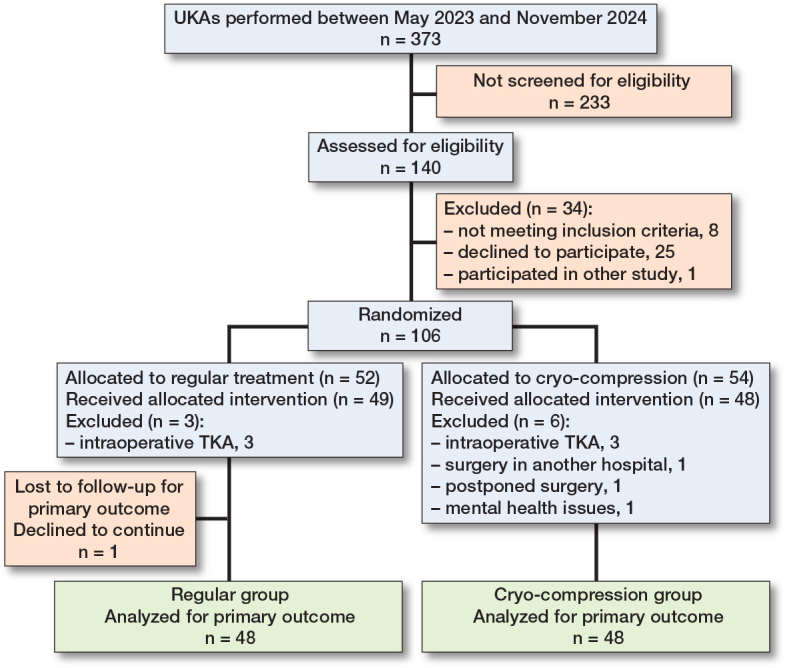
Flowchart of patient exclusion and dropout rate. TKA: total knee arthroplasty. UKA: unicompartimental knee arthroplasty.

## Results

Between May 2023 and November 2024, 373 individuals had a UKA of whom 140 patients were screened for eligibility. 106 participants were randomized to the regular group (n = 52) or cryo-compression group (n = 54). 9 participants were excluded after randomization: 6 due to intraoperative conversion to TKA, and 3 for other reasons (surgery at another hospital, surgery postponed, and mental health issues). The 6-week follow-up period ended in January 2025. During follow-up, 1 participant withdrew due to group dissatisfaction. The Figure shows an inclusion and exclusion flowchart.

### Baseline characteristics

Baseline characteristics were comparable between the groups (Table 1).

**Table 1:** Baseline characteristics

Item	Regular group n = 49	Cryo-compression group n = 48
Patient-related characteristics		
Sex, n		
Men	22	27
Women	27	21
Age, mean (SD)	67 (7.1)	67 (7.7)
Body mass index, mean (SD)	28 (3.9)	28 (4.3)
ASA-score, n		
I	9	8
II	32	34
III	8	6
IV	0	0
Knee compartment, n		
Medial	46	41
Lateral	3	7
Kellgren–Lawrence score, n		
1	0	0
2	1	0
3	22	18
4	26	30
TKA/UKA contralateral knee, n		
No	32	34
Yes	17	14
Surgery-related characteristics		
Analgesics schedule, n		
Normal	31	33
No NSAIDs	18	15
Other	0	0
Length of stay, hours, median (IQR)	27 (21–31)	26 (16–29)

ASA: American Society of Anesthesiologists; TKA: total knee arthroplasty; UKA: unicompartmental knee arthroplasty.

### Primary outcome

The between-group difference in the mean NRS pain-at-rest score at 6 weeks, adjusted for baseline NRS pain at rest, showed no difference (0.1, CI –0.6 to 0.9), with a mean score of 1.6 (CI 1.1–2.1) in the cryo-compression group and 1.8 (CI 1.3–2.3) in the regular group (Table 2). 1 participant in the regular group had a missing NRS pain score at 6 weeks due to withdrawal.

**Table 2:** Pain, function, and quality of life-related outcomes, measured preoperatively and at 6 weeks with between-group comparisons. Values are EMMs with (95% confidence interval)

Item	Regular group (n = 49)	Cryo-compression group (n = 48)	Between-group difference	P value
Primary outcome				
NRS pain at rest ^**a**^				
Preoperatively	4.3 (3.6 to 5.0)	5.5 (5.0 to 6.1)		
6 weeks postoperatively	1.8 (1.3 to 2.3) ^**b**^	1.6 (1.1 to 2.1)	0.1 (–0.6 to 0.9)	0.7
corrected for preoperatively				
Secondary outcomes				
Pain (other than NRS at rest)				
NRS pain during loading				
Preoperatively	6.9 (6.4 to 7.5)	7.7 (7.4 to 8.1)	–0.8 (–1.6 to 0.1)	Time: < 0.001
6 weeks postoperatively	2.4 (1.9 to 3.0) ^**b**^	3.0 (2.4 to 3.6)	–0.6 (–1.6 to 0.5)	Group: 0.01
Change	4.5 (3.6 to 5.4)	4.7 (3.9 to 5.6)	–0.2 (–1.2 to 0.7)	Time × group: 0.6
KOOS–pain				
Preoperatively	45 (40 to 49)	40 (37 to 44)	4.3 (–3.8 to 12)	Time: < 0.001
6 weeks postoperatively	73 (68 to 77) ^**b**^	70 (65 to 75)	2.6 (–6.0 to 11)	Group: 0.2
Change	–28 (–36 to –20)	–30 (–38 to –22)	1.8 (–6.4 to 10)	Time × group: 0.7
KOOS–symptoms				
Preoperatively	60 (55 to 64)	56 (51 to 60)	4.3 (–3.8 to 12)	Time: < 0.001
6 weeks postoperatively	72 (68 to 75) ^**b**^	69 (65 to 74)	2.2 (–5.3 to 9.7)	Group: 0.1
Change	–12 (–19 to –4.4)	–14 (–21 to –6.5)	2.1 (–5.5 to 9.7)	Time × group: 0.6
Function				
OKS				
Preoperatively	34 (31 to 36)	36 (34 to 38)	–2.3 (–6.1 to 1.5)	Time: < 0.001
6 weeks postoperatively	28 (26 to 30) ^**c**^	28 (26 to 31)	–0.2 (–4.6 to 4.1)	Group: 0.3
Change	5.6 (2.1 to 9.0)	7.6 (4.3 to 11)	–2.1 (–5.6 to 1.5)	Time × group: 0.3
KOOS–ADL				
Preoperatively	50 (45 to 55)	43 (39 to 47)	7.3 (–1.3 to 16)	Time: < 0.001
6 weeks postoperatively	79 (75 to 83) ^**b**^	76 (72 to 79)	3.5 (–3.6 to 11)	Group: 0.02
Change	–29 (–36 to –21)	–32 (–40 to –25)	3.9 (–3.7 to 11)	Time × group: 0.3
WORQ				
Preoperatively	46 (42 to 50)	40 (37 to 43) ^**b**^	5.8 (–1.6 to 13)	Time: < 0.001
6 weeks postoperatively	66 (60 to 72) ^**d**^	69 (64 to 74) ^**e**^	–2.9 (–13 to 7.5)	Group: 0.6
Change	–20 (–28 to –13)	–29 (–36 to –21)	8.7 (0.7 to 17)	Time × group: 0.03
ROM (degrees)				
Preoperatively	123 (121 to 125) ^**f**^	123 (120 to 125)	–0.1 (–4.5 to 4.3)	Time: 0.04
6 weeks postoperatively	121 (117 to 124) ^**c**^	120 (116 to 123) ^**b**^	1.0 (–5.3 to 7.2) Group: 0.8	
Change	2.0 (–2.5 to 6.6)	3.1 (–1.5 to 7.8)	–1.1 (–6.0 to 3.8)	Time × group: 0.7
Knee circumference (cm)				
Proximal				
Preoperatively	45 (44 to 46)	43 (42 to 45)	1.6 (–0.7 to 3.9)	Time: < 0.001
6 weeks postoperatively	46 (45 to 47) ^**b**^	44 (43 to 46)	1.2 (–1.2 to 3.6)	Group: 0.1
Change	–0.9 (–1.6 to –0.1)	–1.2 (–2.0 to –0.4)	0.3 (–0.4 to 1.1)	Time × group: 0.4
Mid-patellar				
Preoperatively	43 (42 to 43)	41 (41 to 42)	1.0 (–0.4 to 2.5)	Time: < 0.001
6 weeks postoperatively	44 (43 to 45) ^**b**^	43 (42 to 44)	1.1 (–0.6 to 2.7)	Group: 0.06
Change	–1.2(–2.0 to –0.5)	–1.2 (–1.8 to –0.6)	0.0 (–0.7 to 0.7)	Time × group: 0.9
Distal				
Preoperatively	38 (37 to 39)	37 (36 to 38)	0.7 (–1.0 to 2.4)	Time: < 0.001
6 weeks postoperatively	39 (38 to 40) ^**b**^	39 (38 to 40)	0.8 (–1.2 to 2.8)	Group: 0.3
Change	–1.2 (–2.0 to –0.5)	–1.2 (–1.9 to –0.5)	–0.1 (–0.8 to 0.7)	Time × group: 0.9
Timed Up and Go (sec)				
Preoperatively	11 (9.8 to 11)	11 (9.7 to 11)	0.1 (–1.3 to 1.5)	Time: 0.005
6 weeks postoperatively	9.7 (9.0 to 11) ^**c**^	9.7 (9.0 to 10) ^**b**^	0.0 (–1.4 to 1.4)	Group: 0.9
Change	0.8 (–0.1 to 1.7)	0.7 (–0.4 to 1.8)	0.1 (–1.0 to 1.1)	Time × group: 0.9
Quality of life				
KOOS–QoL				
Preoperatively	30 (26 to 35)	26 (22 to 29)	4.8 (–3.0 to 13)	Time: < 0.001
6 weeks postoperatively	60 (54 to 65) ^**c**^	54 (50 to 59)	5.3 (–4.3 to 15)	Group: 0.04
Change	–29 (–37 to –21)	–29 (–37 to –21)	–0.5 (–9.1 to 8.2)	Time × group: 0.9
EQ5D–5L index score				
Preoperatively	0.63 (0.58 to 0.69)	0.50 (0.43 to 0.58)	0.13 (0.01 to 0.25)	Time: < 0.001
6 weeks postoperatively	0.82 (0.79 to 0.85) ^**b**^	0.79 (0.77 to 0.82)	0.02 (–0.03 to 0.08)	Group: 0.005
Change	–0.19 (–0.25 to –0.13)	–0.29 (–0.39 to –0.19)	0.10 (0.03 to 0.21)	Time × group: 0.008
EQ5D–5L VAS				
Preoperatively	71 (66 to 75)	69 (64 to 73)	2.0 (–6.7 to 11)	Time: < 0.001
6 weeks postoperatively	78 (75 to 81) ^**e**^	78 (74 to 82)	0.1 (–6.4 to 6.5)	Group: 0.7
Change	–7.5 (–12 to –2.7)	–9.5 (–17 to –2.2)	1.9 (–4.5 to 8.4)	Time × group: 0.6

MMs: Estimated Marginal Means; NRS: Numerical Rating Scale; KOOS: Knee Injury and Osteoarthritis Outcome score; OKS: Oxford Knee

Score; ADL: Activities of Daily Living; WORQ: Work, Osteoarthritis and joint-Replacement Questionnaire; ROM: range of motion; QoL: Quality of Life; EQ5D-5L: EuroQol-5 Dimensions-5 Levels; VAS: Visual Analogue Scale.

a The primary outcome, NRS pain at rest at 6 weeks, was analyzed with an ANCOVA corrected for baseline (preoperative values were presented as means with CI). Other outcomes were analyzed using a GEE.

b 1 participant did not complete the questionnaire/test.

c 2 participants did not complete the questionnaire/test.

d 7 participants did not complete the questionnaire.

e 3 participants did not complete the questionnaire.

f 4 participants did not complete the questionnaire/test.

### Secondary outcomes

Regarding oxycodone consumption, after applying the prespecified criterion of ≥ 75% completed entries, data for 9 participants were missing in both groups. During the 6 weeks, the cryo-compression group consumed a median of 3.5 fewer 5 mg oxycodone tablets than the regular group. The proportion using oxycodone 5 mg was lower in the cryo-compression group (18 out of 39) compared with the regular group (27 out of 40), with the most pronounced differences during the first postoperative week(s) (Table 3). Linear regression analysis, adjusted for analgesic schedule and sex, indicated that group allocation was a significant predictor of oxycodone 5 mg consumption (back-transformed β 0.54, CI 0.32–0.91), indicating that the cryo-compression group used 46% less oxycodone 5 mg during the 6 weeks, exceeding the MCID of a 40% decrease in opioid use [30]. This analysis also identified female sex as a significant predictor of oxycodone 5 mg consumption (back-transformed β 1.80, CI 1.07–3.04). After adjusting for analgesic schedule, sex, and length of hospitalization, group allocation was not a significant predictor of oxycodone 10 mg consumption (back-transformed β 0.70, CI 0.46–1.06). Longer length of hospital stay was a significant predictor of oxycodone 10 mg consumption (back-transformed β 1.01, CI 1.00–1.02). At 6 weeks, no difference between groups’ responses to the anchor question regarding improvements in pain were found. Other pain-related outcomes were comparable between groups as well (see Table 2).

**Table 3:** Oxycodone 5 mg and 10 mg consumption and participants using oxycodone

Item	Regular group (n = 40) ^a^		Cryo-compression group (n = 39) ^a^	
	Tablets oxycodone, median (IQR)	Participants using oxycodone, n	Tablets oxycodone, median (IQR)	Participants using oxycodone, n
Oxycodone 5 mg				
Week 1	1 (0–8)	23	0 (0–2.5)	15
Week 2	0 (0–4)	16	0 (0–0)	7
Week 3	0 (0–2.5)	12	0 (0–0)	4
Week 4	0 (0–0)	4	0 (0–0)	1
Week 5	0 (0–0)	3	0 (0–0)	2
Week 6	0 (0–0)	2	0 (0–0)	2
Total	3.5 (0–14.5)	27	0 (0–4)	18
Oxycodone 10 mg				
Week 1	9.5 (5.5–14)	37	6 (2–9.5)	35
Week 2	0 (0–3)	13	0 (0–0)	9
Week 3	0 (0–0)	3	0 (0–0)	1
Week 4	0 (0–0)	1	0 (0–0)	1
Week 5	0 (0–0)	1	0 (0–0)	0
Week 6	0 (0–0)	1	0 (0–0)	0
Total	9.5 (5.5–17)	37	6 (2–10)	35

a 9 participants in both groups did not report and were unable to recall their oxycodone usage.

For the functional outcome WORQ score, improvement was greater in the cryo-compression group compared with the regular group (between-group difference 8.7, CI 0.7–16.6). This did not exceed the MCID of 13 points [21]. At 6 weeks, the groups did not differ in responses to the anchor question regarding improvement in function. Similarly, no differences were observed between groups for the other functional outcomes (see Table 2).

Regarding QoL, the EQ-5D-5L index score improvement was greater in the cryo-compression group than in the regular group (between-group difference 0.10, CI 0.03 to 0.20). This did not reach the MCID of 0.11 points [31]. Other QoL-related outcomes were comparable between groups (see Table 2). For the previously mentioned pain, functional, and QoL-related outcomes, sensitivity analyses of the GEE results using the complete-case subset showed consistency with the primary intention-to-treat analyses, confirming that the data was missing at random.

The cryo-compression group reported a median satisfaction score of 9 out of 10 (IQR 8–10) (mean 8.4, SD 1.3) for the cryo-compression. UKA-related satisfaction score was 8 (IQR 7–9) in both groups, and complication rates were 0 in the regular group and 1 in the cryo-compression group (rehospitalization due to wound leakage). 3 participants reported brace-related discomforts: 2 instances of cold-induced pain and 1 instance of compression-induced pain.

The cryo-compression group used cryotherapy for a median of 40 days (IQR 37–41) out of the instructed 42 and 149.5 sessions (IQR 103–182) out of the instructed 210. Compression was used for a median of 132 sessions (IQR 81–170) out of the instructed 210. The cryo-compression group used cryotherapy via the brace for more days and sessions than the regular group’s use of cold packs (median days 22.5, IQR 9–39, median sessions 46.5, IQR 11–93; both days and sessions P < 0.001).

## Discussion

We aimed to evaluate the effect of 6-week cryo-compression on pain at rest at 6 weeks following UKA compared with standard care and showed no between-group difference in pain at rest. The oxycodone 5 mg consumption during the 6 weeks was lower in the cryo-compression group compared with the regular group, suggesting potential pain benefits. Functional and QoL-related outcomes showed no clinically meaningful differences between groups. UKA-related satisfaction and complication rates were comparable between groups. Brace-related discomfort was minimal, and brace-related satisfaction and protocol compliance were high.

The primary outcome, pain at rest at 6 weeks, was comparable between groups after adjustment for baseline NRS pain at rest. Previous studies in knee arthroplasty patients have predominantly focused their intervention period on the first postoperative week, during which reductions in pain and opioid consumption have been demonstrated [4-8]. In line with our findings, no effect on pain at 6 weeks was observed after a maximum of 1 week of cryotherapy (+ compression) in TKA patients [5-6], nor in the only available study that evaluated cryotherapy specifically in UKA patients [7]. Beyond this first postoperative week, only a few RCTs have evaluated 2-week cryotherapy (+ compression) protocols. Although these studies demonstrated improvements in pain, opioid consumption, or function at 2 weeks postoperatively, these effects were no longer present at 6 weeks [9-11]. This evidence suggests that cryotherapy (+ compression) has no effect on pain at 6 weeks, regardless of intervention duration.

However, when considering opioid consumption, cryo-compression appears to provide benefits, with comparable pain scores between groups. The reduction in oxycodone 5 mg consumption suggests that cryo-compression provides pain relief during the 6 weeks. Oxycodone 5 mg consumption was 46% lower in the cryo-compression group during the 6 weeks, exceeding the MCID [30]. Moreover, fewer participants in the cryo-compression group required oxycodone, particularly during the initial postoperative week, consistent with prior evidence from TKA populations [5-6]. These findings suggest a potential opioid-sparing role for 6-week cryo-compression after UKA, which is especially relevant considering the adverse effects of opioids [3]. However, the absolute median reduction in oxycodone 5 mg consumption was only 3.5 tablets during the 6 weeks.

Functional and QoL-related outcomes showed no clinically relevant benefits from 6-week cryo-compression. Although the WORQ and the EQ5D-5L index score improved significantly more in the cryo-compression group, these did not exceed their MCIDs [21,31]. Brouwers et al. [5] suggested that the WORQ, which assesses physically demanding tasks, may not be sensitive enough to detect recovery differences at 6 weeks in TKA patients. This may apply to this study as well, despite a faster recovery following UKA [1]. The difference in EQ5D-5L index score improvements was likely influenced by baseline imbalances, as both groups reached comparable scores at 6 weeks. Previous studies [4,6] also reported no QoL benefits following 1-week cryotherapy (+ compression) after TKA. Additionally, a few studies on 1 or 2 weeks’ cryotherapy (+ compression) in TKA patients [4,9-10] found improved ROM at 1 or 2 weeks after surgery. However, overall, the evidence for functional benefits is limited, with no studies demonstrating functional benefits at 6 weeks [5-8,11]. The current evidence suggests that cryo-compression does not provide clinically relevant functional or QoL benefits at 6 weeks regardless of duration or patient population.

Brace-related satisfaction was high (median 9.0, mean 8.4), exceeding reports for computer-assisted devices (median 7.5 [32], mean 7.6 [5]), likely reflecting the brace’s usability, portability, and limited burden. Additionally, cryo-compression was safe, with no significant differences in the low complication rates, consistent with a Cochrane review [4]. The 3 reported brace-related minor discomforts did not lead to discontinuation.

Protocol compliance was high for overall treatment duration but lower for daily sessions, probably reflecting the increasing demands of life during later recovery. As ethical constraints prevented withholding cold pack use from the regular group, the substantial use of cold packs in this group, presumably compensating due to awareness of possible benefits, may have underestimated the true effects of cryo-compression. Nevertheless, cryotherapy use remained significantly higher in the cryo-compression group, which also used compression (a component not easily replicable in the regular group).

### Strengths

This study has several strengths, including its randomized design, assessment of multiple outcome domains providing a more comprehensive view of recovery [33], intention-to-treat analysis for most outcomes [27], a single-center setting ensuring standardized care, and assessor blinding.

### Limitations

Participant blinding was not feasible and may have introduced a placebo effect, particularly for subjective outcomes. Differences in baseline scores may have confounded the observed effects. Recall bias could have affected oxycodone consumption data for participants who were contacted later. The single-center design limits generalizability. Additionally, activity levels may have influenced opioid consumption. However, this data was not available in the current study, as collecting patient-reported activity levels over the 6-week period was considered not to be feasible. Lastly, as this study exclusively examined UKA patients and the 6-week results, the effects following TKA and the longer-term outcomes remain unknown.

### Conclusion

6-week cryo-compression following UKA resulted in no between-group difference in pain-at-rest scores at 6 weeks. Oxycodone consumption was lower in the cryo-compression group during this period, suggesting a modest opioid-sparing effect. Functional and QoL-related outcomes showed no clinically meaningful benefits. Cryo-compression was safe, well tolerated, and associated with high patient satisfaction.

*In perspective*, overall, these findings suggest that while 6-week cryo-compression does not improve pain scores, function or QoL, it may have some role in reducing opioid use after UKA.

## Notes

HB: data collection and analysis, and manuscript draft and review. AdV: study conception and design, data collection and analysis, and manuscript review. RB: study conception and design, and manuscript review. All authors read and approved the final manuscript.

The authors would like to thank Marianne de Ronde, Melanie Boer, and Sascha de Haan for their assistance in the practical execution and data entry.

Handling co-editors: Per Henrik Randsborg and Robin Christensen

*Acta* thanks Einar Andreas Sivertsen and other anonymous reviewers for help with peer review of this manuscript.
